# Indications for liver surgery in benign tumours

**DOI:** 10.1007/s10353-018-0536-y

**Published:** 2018-05-22

**Authors:** Margot Fodor, Florian Primavesi, Eva Braunwarth, Benno Cardini, Thomas Resch, Reto Bale, Daniel Putzer, Benjamin Henninger, Rupert Oberhuber, Manuel Maglione, Christian Margreiter, Stefan Schneeberger, Dietmar Öfner, Stefan Stättner

**Affiliations:** 1 Department of Visceral, Transplant and Thoracic Surgery, Medical University of Innsbruck, Innsbruck, Anichstraße 35, 6020 Innsbruck, Austria; 2 Department of Radiology, Medical University of Innsbruck, Innsbruck, Anichstraße 35, 6020 Innsbruck, Austria

**Keywords:** Haemangioma, Focal nodular hyperplasia, Hepatic adenoma, Indication for surgical treatment, Therapeutic strategies

## Abstract

**Background:**

Management of benign liver tumours (BLT) is still object of discussion. Uncertainty still exists about patient selection, details of management, indications for surgical intervention and potential surgery-related complications. The up-to-date strategies for management of the most common benign solid tumours are recapitulated in this article. In addition, recommendations concerning practical issues are presented.

**Methods:**

Available data from peer-reviewed publications associated with the major controversies concerning treatment strategies of solid BLT were selected through a PubMed literature search.

**Results:**

Non-randomized controlled trials, retrospective series and case reports dominate the literature. Conservative management in BLT is associated with low overall morbidity and mortality when applied in an appropriate patient population. Surgical intervention is indicated solely in the presence of progressive symptoms and suspicion of a malignant change. Linking abdominal symptoms to BLT should be interpreted with caution. No evidence is recorded for malignant transformation in haemangiomas and focal nodular hyperplasia (FNH), while a subgroup of hepatocellular adenoma (HCA) is associated with malignancy. Follow-up controls of BLT at 3 and 6 months should be sufficient to prove the stability of the lesion and its benign nature, after which no long-term follow-up is required routinely. However, many questions regarding this topic remain without definitive answers in the literature.

**Conclusion:**

Conservative management of solid BLT is a worldwide trend, but the available literature does not provide high-grade evidence for this strategy. Consequently, further prospective investigations on the unclear aspects are required. Hence, this article summarises practical highlights of therapeutic strategies.

## Main novel aspects

This manuscript summarises current state of the art and practical management recommendations of haemangiomas, FNH and HCA.

## Introduction

The finding of benign liver tumours (BLT) has markedly increased because of recent technical advances in abdominal imaging modalities [1]. Autopsy series reported incidences of up to 50%. BLT are classified into solid and cystic tumours according to features on radiographic imaging. Table 1 gives an overview of benign hepatobiliary lesions. The most common solid BLT are haemangiomas, focal nodular hyperplasia (FNH) and hepatocellular adenoma (HCA), whereas simple cysts represent the most common non-solid lesions [2]. Other incidental imaging findings include atypical cysts, focal fatty sparing and hepatic cystadenoma [3]. In the past years, surgery was advocated for these findings, in view of their uncertain clinical behaviour. In malignant hepatobiliary lesions, liver resection has become widely accepted as the only potentially curative treatment. However, with improvements in imaging, better understanding of causes and histology of BLTs, surveillance has become a valid alternative in most patients. With the increasing discovery of BLT, clinicians are increasingly faced with the need to make therapeutic decisions regarding the management of these tumours [2]. Before 1980, liver resection was associated with mortality rates above 10%. However, in the past decade, overall mortality has decreased to 5% in high-volume centres because of better knowledge of liver anatomy, refinements in surgical techniques and advances achieved in post-operative care [4]. As a consequence, an increasing number of patients with benign lesions are nowadays considered for surgical treatment [5]. Despite the low mortality and morbidity rate after partial liver resection of less than 2% for metastatic disease, there is still discussion regarding the indications for surgical liver resection of benign hepatobiliary lesions [6]. For BLT such as haemangioma, FNH and HCA, surgery may be indicated solely on the presence of progressive symptoms and suspicion of a malignant change [3]. Although malignant transformation of BLT is an uncommon phenomenon, it can occur [7]. In particular, patients with multiple large adenomas have a greater chance for malignant transformation [8]. Many patients present with non-specific abdominal pain in the setting of BLT; nevertheless, combining these symptoms with the tumours is challenging and controversial. The overall indication and utilization of surgery may be subjective and variable. Additionally, increased use of minimally invasive surgical (MIS) approaches may impact the relative use of surgery for BLT [2]. When a patient is considered for surgery, complete information about risks and alternative treatment options should be discussed. Subjective symptoms and impact on daily life are just as important as the outcome data of surgery [9]. Kim et al. demonstrated that the volume of operative procedures for BLT has increased significantly over the past decade [2]. Mezhir et al. identified patients with BLT from an institutional database. A significant increase in the number of BLTs diagnosed over time and a trend toward observation were observed. During the time covered by this study, the percentage of patients who were taken for immediate resection declined in more recent years. The findings suggest that most patients with a BLT can remain subjected to observation with low risk for misdiagnosis, complications or malignant transformation [3]. This article summarises practical highlights and therapeutic management of the most common solid tumours.

## Methods

Available data from peer-reviewed publications associated with this topic were selected through a PubMed literature search. Articles were selected using the indexing terms “benign liver tumours”, “liver neoplasm surgery”, “focal nodular hyperplasia”, “liver haemangioma”, “hepatocellular adenoma”. Only larger series with more than 30 patients with benign lesions were included. Due to lack of data, meta-analysis including descriptive statistics was not possible. The current literature concerning this topic was summarised.

## Results

### Haemangioma

**Fig. 1:**
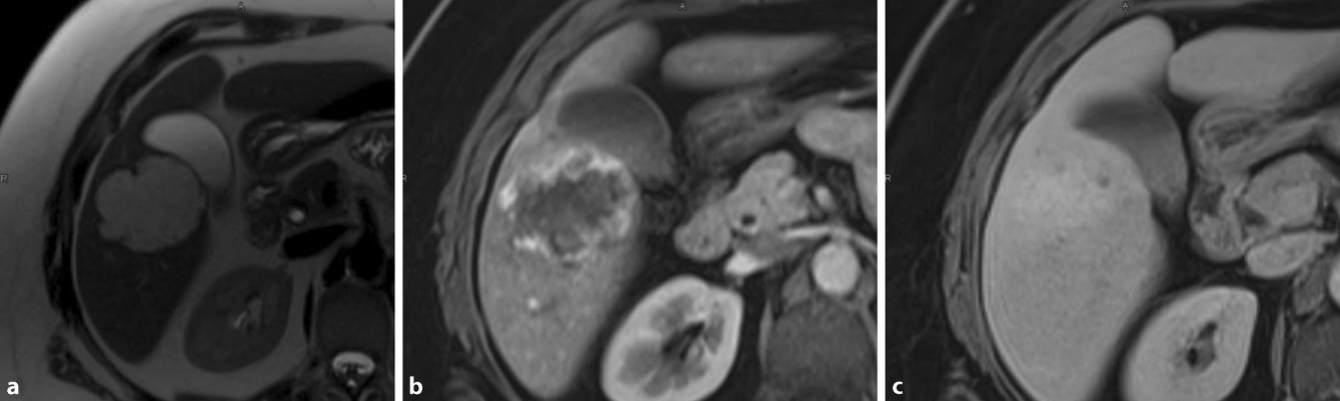
Haemangioma in segment V: axial T2-weighted (**a**), arterial phase (**b**) and late phase approximately 2 min after the i. v. injection of contrast agent (**c**). Typical hyperintense signal in the T2-weighted images and the peripheral enhancement in the arterial phase with nearly isointense presentation in the late phase

Liver haemangiomas are common incidental findings, reflecting a high prevalence within the common population ranging from 1 to 20% [7]. Originating from the mesodermal layer, these lesions represent a congenital non-neoplastic hamartomatous proliferation of vascular endothelial cells. Macroscopically, these tumours are well-circumscribed hypervascular lesions with good compressibility; hence, no classical evidence for malignant potential is given. The majority of haemangiomas are of the cavernous type, representing the most common BLT. These lesions have been reported in up to 7% in autopsy studies [10]. Unlike the less common capillary-type haemangiomas, which are generally smaller in size, multiple and most commonly asymptomatic cavernous haemangiomas can grow to a large size and may become symptomatic. Giant haemangiomas are defined as those measuring ≥5 cm, while hypergiant hepatic haemangiomas are defined as those which are larger than 10 cm in size [11]. These lesions are more frequent in women (female:male ratio = 5:1), with a mean age at diagnosis of 50 years [12]. The certainty of diagnosis sustained by high-quality non-invasive imaging represents still an important step. Transabdominal ultrasound (US) is diagnostic in approximately two thirds of cases [13]. However, axial imaging may be undertaken and is crucial prior to therapy. Haemangiomas tend to be hypodense on native computed tomography and show centripetal contrast uptake. These characteristics are of important value in differentiating haemangiomas from metastases [14]. Magnetic resonance imaging (MRI) may also be of importance in difficult cases. As shown in Fig. 1, typical features include high signal intensity on T2-weighted series and discontinuous nodular peripheral enhancement. Diagnostic biopsy to differentiate giant haemangiomas from malignant lesions should in general be discouraged. On the one hand there is a reported risk of haemorrhage of up to 0.28% [15], on the other hand the risk of needle track seeding and intra-abdominal dissemination of a potentially curable malignancy is of clinical importance [10]. Cavernous haemangiomas occur more frequently in the right liver, an association with oral contraceptives (OCPs) still remains controversial [10]. Current evidence reveals an uncomplicated natural history of these lesions.

Linking abdominal symptoms to liver haemangiomas should be interpreted with caution. Eventual symptoms unrelated to an incidentally detected haemangioma should be clarified and alternative causes excluded (e. g. gallstones, gastroduodenitis, orthopaedic affection). Patients with large lesions may present abdominal pain, due to tension of Glisson’s capsule, compression of local structures, intra-lesional thrombosis and infarction or haemorrhage. The risk of haemorrhage seems to be less than 1% [16] and for decision making probably negligible. A group of 437 patients from a single institution were analysed with regard to a diagnostic algorithm, the indications for surgery and observation in BLT. Observation of patients with haemangiomas for a median of 32 months revealed that these vascular lesions remain stable in size without risk of malignant transformation [6]. Despite reaching large dimensions, spontaneous rupture of a giant haemangioma is exceptional. Fewer than 50 cases of spontaneous rupture have been reported in the literature [17]. Traumatic rupture is a recognised complication, but only a handful of cases have been described [18]. Kasabach–Merrit syndrome causes thrombocytopenia and consumptive coagulopathy in association with large haemangiomas. Platelet trapping in the haemangioma is thought to result in activation of platelets and the clotting cascade, resulting in consumptive coagulopathy [19]. However, the optimal approach and the indication for resection of haemangiomas remain controversial. Today, surgery is the most effective therapeutic strategy for the definitive treatment of liver haemangiomas [10]. Indications for surgical interventions are suspected malignant transformation or misdiagnosis, symptoms correlating with a growing abdominal mass and consecutive complications, such as haemorrhage, infarction, rupture or Kasabach–Merritt syndrome [20]. Additionally, full blood tests should be performed. Abnormalities may indicate haemorrhage, infarction, neoplasia or other causes, but in most cases, liver biochemistry is normal [10].

Considering a benign and uncomplicated natural history for the majority of haemangiomas, non-operative management might be the right approach for the majority of patients. The risk of potential complications should be carefully weighed against operative risks. Surgery should therefore be reserved as salvage treatment after exclusion of other causes and open discussion with the patient [10]. Alternatively, transarterial chemoembolization (TACE) with the antineoplastic antibiotic bleomycin has been used as a sclerosing agent in the treatment of vascular malformation and haemangiomas. One study suggested that bleomycin, a cytotoxic agent, may inhibit haemangioma growth by inhibiting neovascularization [21], but using a chemotherapeutic agent for a benign disease is at least worth a proper multidisciplinary discussion. Transarterial embolisation with polyvinyl particles is also described in a small series [22], but the success rate seems rather questionable as other studies reported no positive effect. The static nature of giant haemangiomas makes further follow-up unnecessary [23]. Pregnancy does not pose a higher risk, although single cases with complicated courses are published. Furthermore, enlargement during pregnancy is suggested to be caused by hormonal influence [24]. Due to lack of evidence, individual treatment is mainly used in these cases. Surgery still remains a possible option in symptomatic and growing lesions. OCPs and other oestrogen-containing preparations can be further administered, but close controls at 6 and 12 months are recommended [6]. Patients with abdominal compressive symptoms may be more likely to derive benefit from surgery than patients with unspecific abdominal discomfort. However, symptoms persist in 25% of patients following resection on haemangiomas and therefore a clear communication with the patients is essential.

### Focal nodular hyperplasia

FNH is a non-malignant hepatic tumour without vascular origin, often diagnosed incidentally on radiologic imaging. Autopsy series reported that 8% of non-haemangiomatous lesions were FNH, representing 66% of all benign non-haemangiomatous lesions [25]. FNH occurs mostly solitary (80–95%) and is usually less than 5 cm in diameter. It is seen throughout all age spectra but mainly in women (female:male ratio = 9:1) [26]. FNH has various synonyms: solitary hyperplastic nodule, hepatic hamartoma, focal cirrhosis, hamartomatous cholangiohepatoma and hepatic pseudotumor. Previously FNH was considered to be either a hamartoma, a neoplasm, a response to ischemia or injury, or a focal area of regeneration. Nowadays it is generally described as a hyperplastic, regenerative response to hyperperfusion with characteristic anomalous arteries found in the centre of these nodules [26, 27]. Main characteristics are sharp margins, nodular architecture, abnormal vessels, proliferation of bile ducts and a central stellate scar which is found in about 50% of cases [28]. Non-classical forms of FNH lack either the typical nodular architecture or vascular malformations, but always present bile ductular proliferation. FNH might also be associated with hereditary haemorrhagic telangiectasia, hepatic haemangiomas, and other vascular malformations [25]. It has no malignant potential and is rarely symptomatic.

**Fig. 2:**
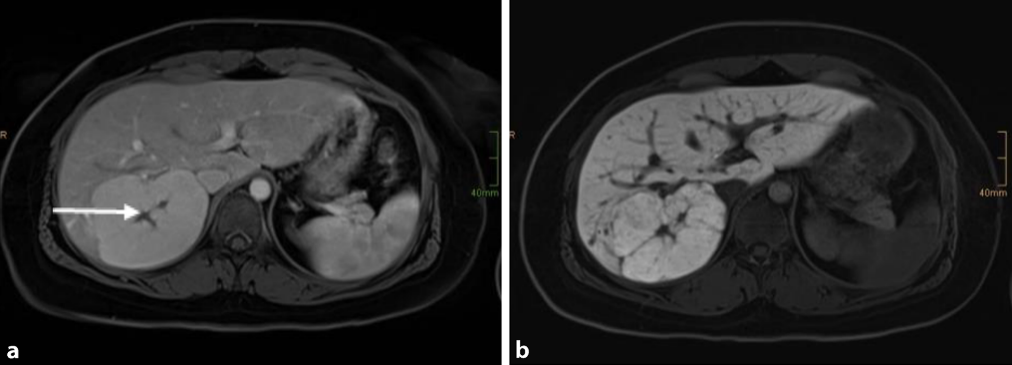
FNH: axial MRI in the late arterial phase (**a**) with Primovist® (Bayer Schering Pharma, Berlin, Germany) shows a large, slightly hyperintense lesion (*arrow*), with a hypodense centre, in the right liver, consistent with a central scar (*arrowhead*) [9]. The hepatobiliary phase (**b**) images show a strong uptake of Primovist, which is a typical sign of FNH

As shown in Fig. 2, the radiological diagnosis of FNH is often made on the basis of the detection of a central scar. However, this finding might be missing in half of the patients. On the other hand, a central scar may also be found in patients with fibrolamellar hepatocellular carcinoma, hepatic adenoma or intrahepatic cholangiocarcinoma. This limitation applies to all cross-sectional imaging studies. In angiography, the characteristic stellate appearance is demonstrated in only 33% of patients; moreover, FNH may even be avascular in 10% of cases. Therefore, the diagnosis of FNH is sometimes only achieved by use of several complementary imaging techniques. In patients with unclear diagnosis, a biopsy or surgical resection may be needed to achieve complete pathohistological workup. Laboratory tests are most often normal and therefore not helpful in the diagnosis. Clinical symptoms directly attributable to FNH are infrequent and therefore difficult to link to the tumour. Acute haemorrhage, necrosis or infarction are extremely rare [29]. Thus, FNH should be managed similarly to haemangiomas, with very strict indications for surgery.

The natural history of FNH is uneventful, lacking complications and changes over time. However, enlargement in the setting of OCPs and during pregnancy have been reported [30]. To date, there is no evidence for malignant transformation of FNH [28, 31–33]. According to the majority of reports and the authors experiences, patients with FNH should be managed conservatively [6, 31, 34, 35]. Follow-up controls at 6 months should be sufficient to prove the stability of the lesion and its benign nature, after which no long-term follow-up is routinely required. Surgery should be only considered for symptomatic FNH lesions, or highly suspicious lesions where malignancy cannot be ruled out with modern imaging or even biopsy. Numerous reports regarding FNH during pregnancy have been published, making an association with endogenous and/or exogenous oestrogens very likely. As for haemangiomas, close controls at 6 and 12 months are recommended. Small lesions do not appear to pose a significant risk to a successful pregnancy, although observation is strongly recommended and resection may be prudent for large (>8 cm) FNH lesions [6]. Liver-directed therapies are only reported in small series, hence no evidence-based recommendation can be made and surgery remains the preferred therapy if properly indicated.

### Hepatocellular adenoma

**Fig. 3:**
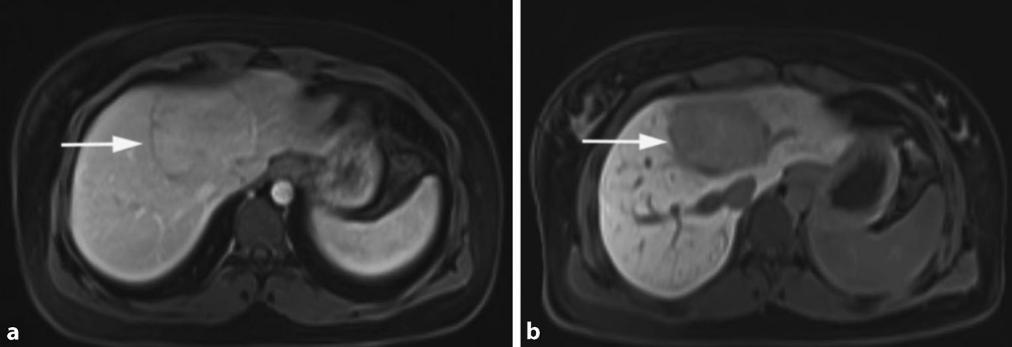
HCA: axial venous phase MRI with Primovist® (Bayer Schering Pharma, Berlin, Germany) shows an only slightly hyperintense lesion (*arrow*) in the centre of the liver (**a**) with a marked capsule. In the hepatobiliary phase no uptake of contrast agent is seen within the lesion (*arrow*), which is further consistent with HCA (**b**) [9]

HCA is a rare, solid and benign liver tumour of presumable epithelial origin. The estimated prevalence is about 0.004%. These lesions occur mostly in women of childbearing age and seem strongly associated with the use of OCPs and oestrogens [36]. Women older than 30 years taking OCPs for longer than 5 years have the highest risk levels. A causal role for hormone activity in HCA growth is suggested by data linking adenoma regression to the cessation of OCP use and, vice versa, growth associated with pregnancy [37]. Hepatic adenomas appear as single or multiple lesions and may occasionally reach a size larger than 20 cm. Hepatic adenomatosis is an equally uncommon condition in which >10 nodules develop in the absence of classical risk factors such as OCPs. There is a strong association seen with glycogen storage disease. As shown in Fig. 3, HCA is usually detected by imaging, typically US or multi-phase contrast-enhanced imaging studies such as CT or MRI scans. The significance of making a specific diagnosis is that, unlike haemangioma and FNH, HCA has a small but meaningful risk of progressing into malignancy. Although imaging provides supportive information, a definitive diagnosis of hepatic adenoma requires biopsy of the tissue [38]. The introduction of a new classification system for HCA helps clinicians in tailoring the treatment. Patients are stratified according to imaging criteria, expression profile of associated immunohistochemical markers or molecular findings. This classification includes hepatocyte nuclear factor 1α-inactivated HCA (H-HCA 30–35%), β‑catenin-mutated HCA (b-HCA 10–15%), inflammatory HCA (I-HCA 50%) and a subgroup of unclassified cases (less than 10%) [39]. The b‑HCA group appears to be related to hepatocellular carcinoma [38].

Historically, HCAs were treated with a watch and wait policy and the unselective recommendation to avoid OCPs. Surgical intervention was preferred for larger lesions—the “5 cm rule”—due to an expected higher risk of malignant transformation. However, current management options may also include minimally invasive techniques like radiofrenquency ablation (RFA) and transcatheter arterial embolisation (TAE). New molecular insights have shown that b‑HCA and I‑HCA are prone to malignant degeneration especially if the size reaches >5 cm. In these instances, invasive treatment is recommended [38]. According to van Bieze et al., the risk of haemorrhage in HCA is the highest among BLTs. Risk factors for bleeding of HCA include a diameter of 35 mm or more, visualisation of lesional arteries, location in the left lateral liver and exophytic growth [40]. Women with HCA who are pregnant should be closely monitored for HCA size due to the tendency of the lesion to grow [37]. Treatment of HCA during pregnancy may be indicated when the lesion shows signs of growth or bleeding. Whether some subtypes are more prone to complications during pregnancy is not known, mainly because the majority are diagnosed non-invasively. The choice of the right management, i.e. surgery, RFA, TAE or follow-up in pregnancy is often a matter of debate. Surgery of lesions located at the periphery of the liver can be performed safely within the first or second trimester and will probably be indicated by the size and location of the lesion. Given the increased risk of haemorrhage in larger HCAs, a pre-emptive treatment strategy before pregnancy is recommended [41]. If HCA is discovered during pregnancy, the second trimester would be the optimal moment for invasive treatment, as anaesthesia is well tolerated at this stage and the foetus is not grown enough to interfere with liver surgery.

One of the major discussions involves the clinical application of the molecular subclassification in the diagnosis and treatment of adenomas, balancing the risk of an invasive liver biopsy with benefits of an individualised therapy. When a lesion is >5 cm, OCPs should be stopped and MRI performed after 6 months. If the lesion has decreased to <5 cm, definitive signs of an H‑HCA should be ruled out. If H‑HCA is subsequently identified, therapy can be less aggressive because the risk of malignant progression is very low. Follow-up should be performed, initially every 6 months, and if the lesion shows no further alteration, follow-up can be stopped or repeated yearly until menopause [42]. For small lesions (<5 cm) categorised as I‑HCA, management should not differ. However, a biopsy to exclude β‑catenin mutation needs to be discussed as there is no reliable imaging characteristic to diagnose b‑HCA non-invasively. For larger lesions (>5 cm) with a β-catenin mutation and additional risk factors such as male sex, steroid use, glycogen storage disease or underlying viral hepatitis, treatment is clearly recommended. Mainstay of treatment is liver resection and complete pathohistological workup; in selected cases, RFA or TAE may be used. For hepatic adenomatosis, orthotopic liver transplantation is a valid option [43]. We strongly advocate multidisciplinary discussion of all cases in specialised teams to create the best individual management plan, especially in childbearing females.

## Conclusion

Most patients with a BLT can be observed with a low risk of misdiagnosis, complications or malignant transformation. In the past years, a trend towards conservative management could be observed [3]. Patients indicated for resection include those with symptomatic tumours or lesions in which malignancy cannot be excluded. The management for adenomas is based primarily on imaging criteria, expression of associated immunohistochemical markers and molecular findings. Special attention is advocated for the b‑HCA subtype with increased risk of malignant transformation.

The risk of potential complications and the severity of symptoms need to be weighed against surgical risk. Minor liver resection for the treatment of a wide range of BLT is associated with low morbidity (9%) and virtually no mortality in centres with a high volume of patients. The presence of comorbidity, prolonged surgical time and incomplete resections are associated with major morbidity. Besides parenchymal-sparing approaches with close margins, laparoscopic resections provide a benefit in terms of pain reduction and comfort, especially in the long term [44]. For all three kinds of lesions, if follow-up controls are indicated, 3 and 6 months should be sufficient to prove the stability of the lesion and its benign nature, after which no long-term follow-up is routinely required.

**Table 1:** Common benign liver tumours

Solid lesions	Haemangioma, FNH, HCA, angiomyolipoma, hepatic lipoma, mesenchymal hamartoma
Cystic lesions	Hepatic cyst, hepatobiliary cystadenoma
